# Improving the quality of life in a breast cancer patient and caregiver: Protocol for the application of the integrative medical service model

**DOI:** 10.1097/MD.0000000000032244

**Published:** 2022-12-16

**Authors:** Moon Joo Cheong, Won-Bae Ha, Han-Baek Cho, Un-jong Choi, Hyeon-Jun Woo, Yun-Hee Han, Hyung Won Kang

**Affiliations:** a Rare Diseases Integrative Treatment Research Institute, Wonkwang University Jangheung Integrative Medical Hospital, Anyang-myeon, Jangheung-gun, Jeollanam-do, Republic of Korea; b Department of Korean Medicine Rehabilitation, College of Korean Medicine, Wonkwang University, Iksan-si, Jeollabuk-do, Republic of Korea; c Department of Korean Medicine Obstetrics & Gynecology, College of Korean Medicine, Wonkwang University, Iksan-si, Jeollabuk-do, Republic of Korea; d Department of Surgery, Wonkwang University School of Medicine, Iksan-si, Jeollabuk-do, Republic of Korea; e Department of Korean Neuropsychiatry Medicine, College of Korean Medicine, Wonkwang University, Iksan-si, Jeollabuk-do, Republic of Korea.

**Keywords:** breast cancer, caregiver, integrative medical service model, quality of life

## Abstract

Patients with chronic diseases require ongoing treatment, and caregivers face financial burdens as well as psychological and physical difficulties. However, the current healthcare system does not provide adequate systems or services to address the difficulties that patients and caregivers face.

**Purpose:**

The purpose of this study was to conduct an observational case study in order to evaluate and improve the application of an integrative healthcare service model developed for distress management and improved quality of life in breast cancer (BC) patients and caregivers.

**Method:**

The integrative healthcare service model was intensively applied to a patient-caregiver pair in this observational study. This was followed by gathering feedback from participants and experts, as well as reflecting on the content of the feedback in order to improve the model further.

**Results:**

This study will then modify and improve the program with feedback and provide integrative medical services to a BC patient and caregiver.

**Conclusion:**

This study used the BC patients’ pain management and quality of life enhancement model, aiming to provide basic data for the establishment of a healthcare service system for patients suffering from chronic pain due to diseases such as BC by systematically integrating previously applied interventions into the current healthcare system and soliciting feedback from patients and caregivers.

## 1. Introduction

Chronic diseases, such as heart disease, cancer, diabetes, and stroke, and geriatric diseases, such as arthritis, need to be approached from the perspective of integrative healthcare services rather than from a general therapeutic perspective.^[1]^

Chronic diseases require continuous treatment for patients, and caregivers are faced with financial burden and psychological and physical difficulties.^[2]^ However, the current healthcare service system fails to provide sufficient systems or services to address such difficulties encountered by patients and caregivers.^[3,4]^ Moreover, medical experts agree that there is a compelling need for developing and providing diverse forms of healthcare services. This would allow for interventions for patients with any of the 4 chronic diseases as well as geriatric diseases and their caregivers, as these diseases require continuous treatment and management.^[5]^

Against this backdrop, we previously conducted integrative healthcare service model development research at the national level, with patients with the 4 major severe diseases and geriatric diseases as the target population.^[6]^ Empirical feedback from patients and caregivers is essential for enhancing the on-site accessibility of the proposed integrative healthcare service model and further developing it into a well-established model that provides practical contributions to the well-being of patients and their caregivers. In particular, patients with (hereinafter “breast cancer [BC] patients”) face conflicts with caregivers as well as drug misuse and abuse due to the inevitable deterioration of quality of life (QoL) while undergoing treatment. However, on-site implementation of appropriate management or education is fraught with considerable difficulties.

Therefore, it was considered necessary to conduct a case study to provide a basis for improving the healthcare service system. Such a case study should embrace the application of the integrative healthcare service model developed for the distress management and QoL enhancement of BC patients and their caregivers. In this context, the present study intensively applied an integrative healthcare service model to a single BC case involving a patient-caregiver pair. Feedback from participants and experts was collected and analyzed in order to further improve the model.

## 2. Method/design

### 2.1. Methodology

This observational study will apply the integrative healthcare service program developed for pain management and QoL enhancement of BC patients to a BC patient-caregiver pair. Quantitative and qualitative feedback from diversified perspectives will be obtained to facilitate the application and refinement of the program in practical settings by researcher and clinical research cordinator. In order to obtain research consent, the research manager will explain the procedure to the participants in the research progress, compensation, and withdrawal. In additional, Collect, share and maintain personal information about potential and registered participants. In order to protect confidentiality before, during and after trial, the Research Officer will store all data on the Research Officer’s personal computer, act in accordance with the management of the IRB, and fulfill his responsibilities. All data will be discarded 3 years after the end of the study.

### 2.2. Participants

#### 2.2.1. Patient.

1) Inclusion criteria:
  (1) Female, aged 20 to 65 years
  (2) BC patient before the initiation of 4-week adjuvant radiation therapy after BC surgery
  (3) Ability to read and complete the questionnaire
  (4) Ability to understand the purpose of this research and agree in writing to participate in the study
2) Exclusion criteria
  (1) A history of major psychiatric disorders such as those included in the DSM-5 diagnosis of psychiatric symptoms, that is, schizophrenia spectrum disorder, delusional disorder, bipolar disorder, and alcohol/substance use disorder
  (2) Affected by a chronic disease that might influence the study results
  (3) Participation in other clinical trials within the past month
  (4) Inability to communicate normally due to dementia or mild cognitive impairment
  (5) Inability to communicate and limited ability to read and complete the questionnaire
  (6) A medical condition that, as judged by the principal researcher, hinders participation in the study

#### 2.2.2. Caregiver.

1) Inclusion criteria
  (1) Caregiver aged 20 to 65 years designated by the enrolled patient
  (2) Voluntarily consented to participate in this study (The term “caregiver” refers to an adult member of family or a trusted person who assists the patient in carrying out activities of daily living)
2) Exclusion criteria
  (1) Communication problems associated with serious psychiatric conditions, intellectual disability, mood disorders, or other cognitive problems that, as judged by the principal researcher, may hinder participation in the study

### 2.3. Sample size

In this case study, a patient–caregiver pair was provided with healthcare services, and the participants offered their respective feedback. According to Boddy,^[1]^ the sample size in a case study is determined based on the situation and the scientific paradigm of the research being conducted; moreover, a single case study involving a single participant can generate great insight. Therefore, the present study selected a total of 2 samples (1 patient-caregiver pair).

## 3. Research details

### 3.1. Integrative healthcare service model

The “integrative healthcare service model for QoL enhancement of patients with BC and their caregivers” was used in this study. The proposed model has the following features.

BC patients are diagnosed and treated by medical staff who are trained in both western and oriental medicine (Korean traditional medicine) and are unique to the Korean healthcare system. Following integrative services are a variety of complementary and alternative services that have been prioritized from among the physical pain management interventions derived from the literature and expert opinions.

These integrative healthcare services are supplementarily provided, not only to patients, but also to their caregivers. Caregivers are primarily given information about the disease’s characteristics by the medical staff and the coordinator in charge of integrative healthcare services. In the case of BC, the importance of QoL-related supplementary treatments and interventions to address symptoms such as fatigue, general weakness, pain, depression, anxiety, nausea/vomiting, and hot flushes is highlighted. First, medical staff members describe the disease and treatment options. Then, caregivers are given additional information about the disease via video clips that illustrate the lives of BC patients. Furthermore, patients and caregivers participate in intervention programs that allow them to work together, such as aromatherapy, yoga exercise, make-up program, nutrition education, and menopause education, which are part of a broader range of integrative services designed to improve patient-caregiver relationships and better understand the patient. Finally, caregivers are provided with information on financial aid, including insurance, and on the networks that connect healthcare providers with the community, so that the support can be extended to daily living after completion of the program.

### 3.2. Integrative healthcare service program

#### 3.2.1. Program composition.

This subsection describes the integrative healthcare service program, which is an optional complementary and alternative medicine service.

The program is based on an existing integrative healthcare service model developed as part of the Ministry of Health and Welfare’s integrated healthcare support project to improve the QoL of BC patients and caregivers. In a study using this model, the research team improves the program based on feedback gathered during each session in consultation with the medical staff to avoid problems that may arise during clinical application. The program’s specifics are provided below.

##### 3.2.1.1. Healthcare services.

Based on the current course of the disease, the patient receives consultation and prescriptions for drugs and pain injections. The oriental medicine hospital then provides services, beginning with a consultation with the medical staff and continuing with treatments such as acupuncture, Chuna therapy, and physiotherapy.

##### 3.2.1.2. Integrative services.

(1) Patient program: Breathing meditation, exercise, treatments, and interventions applying various media for pain management and emotional control.
  • The breathing meditation program is either guided by an expert or self-directed using a 5-minute video clip.
  • The exercise program focuses on pain management, and materials (media) for later use can be provided when done in conjunction with the caregiver. This program aims to provide a variety of experiences designed to improve the patient and caregiver’s mind-body health and QoL.
  • Treatments and interventions using various media include yoga exercise, exercise using psychological intervention, make-up program, and laughter therapy. Multifaceted media interventions give people time to forget about their pain and focus on something else. Furthermore, involving the caregiver in a program increases their understanding of the disease, which increases the possibility of improving their relationship at the same time. The corresponding expert or specialist oversees each component of the program.

##### 3.2.1.3. Special feature.

Program details are adjusted every week based on the expert, patient, and caregiver opinions and feedback.

### 3.3. Program implementation plan

Consultation and treatment are provided in western and oriental hospitals, and integrated services are provided in hospital safe places at fortnightly intervals in the form of an 8-session program administered in the presence of medical staff. The duration of each session is limited to 100 minutes. Table 1 is an example of the program implementation.

**Table 1:** Integrative Medical Service program for the breast cancer patient–caregiver pair.

Session		Breast canter patient and caregiver program: pain management and QoL								Duration
W1	1	P	Consultation (O↔W) 20 min	Questionnaire completion		Breathing meditation (5 min)	Aromatherapy (30–40 min)		Feedback gathering	≤ 100 min
		C	Questionnaire completion and psychological testing				Breathing meditation (5 min)	Physiotherapy (Healing center) (30 min)	Feedback gathering	
	2	P	Consultation (O↔W) 20 min		Breathing meditation (5 min)		Yoga for pain management		Feedback gathering	≤ 80 min
		C	Psychological test interpretation (25 min)		Breathing meditation (5 min)				Feedback gathering	
W2	3	P	Consultation (O↔W) 20 min				Make-up program (40 min)		Feedback gathering	≤ 80 min
		C					Make-up program (40 min)		Feedback gathering	
	4	P	Consultation (O ↔W) 20 min				Nutrition education (20 min, P + C)	Physiotherapy (Healing center) (15 min)	Feedback gathering	≤ 80 min
		C							Feedback gathering	
W3	5	P	Consultation (O↔W) 20 min		Breathing meditation (5 min)		Medication education (20 min, P + C)	P + C program I Mind exercise	Feedback gathering	≤ 80 min
		C			Opinion gathering (25 min)				Feedback gathering	
	6	P	-		Consultation (O↔W) 20 min		Menopause education I (20 min, P + C)	Laughter therapy (30 min)	Feedback gathering	≤ 80 min
		C			Opinion gathering (20 min)				Feedback gathering	
W4	7	P	-		Consultation (O↔W) 20 min		Menopause education II (20 min, P + C)	Program (30 min) Physiotherapy (Healing center) (20 min)	Opinion gathering	≤ 80 min
		P			Opinion gathering (20 min)					
	8	P	Questionnaire completion (10 min)		Consultation (O↔W) 20 min		Laughter therapy (40 min)	Questionnaire completion Opinion gathering		≤ 80 min
		C			Counseling (60 min)					

C = caregiver, O = oriental medicine, P = patient, W = week, W = western medicine, QoL = quality of life.

### 3.4. Assessment items

#### 3.4.1. Sociodemographic and epidemiological characteristics of patient and caregiver.

The following sociodemographic and epidemiological variables were considered: patient’s sex, age, marital status, educational level, health insurance coverage type, assumption of personal care costs, primary caregiver, duration of illness, person who settles hospital bills, religion, occupation, self-rated economic status, last menstrual period, type of surgery, date of surgery, age at diagnosis, stage at diagnosis, chemotherapy, adjuvant radiation therapy, targeted therapy, ovarian suppression therapy, start date of hormone replacement therapy, age at the start of tamoxifen intake, menstrual status before tamoxifen administration, current menstrual status, tamoxifen discontinuation (Y/N), and after-tamoxifen stress clinic or psychiatric care experience (Y/N).

#### 3.4.2. Primary outcome for patient and caregiver.

##### 3.4.2.1. Health-related quality of life (HRQoL) assessment tool.

1. HRQoL measure: EuroQoL-5 Dimension 5-Level (EQ-5D-5L)

The EuroQol Group introduced the euroqol-5 dimension 5-level (EQ-5D-5L) in 2009 as a new QoL measurement tool, extending the 3 domains of the EQ-5D-3L to 5 to compensate for its shortcomings. While the EQ-5D-3L describes 243 health states, the EQ-5D-5L describes 3125 health states, reducing the ceiling effect of the EQ-5D-3L, thereby increasing the reliability and sensitivity of the EQ-5D-3L and enriching technological resources. In a study comparing the EQ-5D-3L and EQ-5D-5L in cancer patients in a Korean hospital, the EQ-5D-5L was found to have a lower ceiling effect, higher validity, and similar test-retest reliability compared with the EQ-5D-3L.^[6]^ The EuroQol Group also introduced a Korean version of the EQ-5D-5L, and published the results.^[7]^

1. EuroQol Visual Analogue Scale (EQ-VAS)

The Euroqol visual analogue scale (EQ-VAS) is a rating scale that records self-rated health state on a 20-cm-long vertical visual analogue scale. The scale assigns top-to-bottom numeric values ranging from 100 (best imaginable health state) to 0 (worst imaginable health state). Scores obtained provide a clear order of health outcomes as well as information on the intensity of preferences. As an inherent drawback of a rating scale, the EQ-VAS is subject to measurement bias. Rating scales are usually recommended as an auxiliary tool, and the EQ-VAS was included as an auxiliary tool for evaluating the EQ-5D-5L and EQ-5D-3L questionnaires.

#### 3.4.3. Secondary outcome.

##### 3.4.3.1. Patient outcomes.

1. Core Seven-emotions Inventory-short form (CSEI-s)

The core seven-emotions inventory-short form (CSEI-s)^[8]^ is a 28-item scale consisting of 7 domains that represent basic human emotions in oriental medicine: joy (4 items), anger (4 items), thought (4 items), anxiety (4 items), sorrow (4 items), fear (4 items), and fright (4 items). Each item is rated on a 5-point Likert scale (1 = not at all to 5 = very much so). The scores of 6 emotions excluding joy that surpass the T score (M = 50, SD = 10) are considered to be within the at-risk range – the higher the score, the higher the risk. Specifically, the T score cutoff points are 55 to 60 for caution group, 61 to 65 for risk group, and ≥ 66 for high-risk group. By contrast, lower joy scores indicate a higher risk: 40 to 45 for caution group, 35 to 39 for risk group, and ≤ 35 for high-risk group.

1. Distress Thermometer (Korean Edition-NCCN Guidelines for Distress Management)

Distress was measured using the Korean version of the national comprehensive cancer network (NCCN) distress thermometer (DT),^[9]^ Korean Edition-NCCN Guidelines for Distress Management Version 2.2013.^[10]^ The DT expresses the degree of distress felt in the past week, including the current day, on a rating scale from 0 (no distress) to 10 (excessive stress). The cutoff score is 4 points: mild distress < 4, moderate-to-severe distress ≥ 4. At the same time, the problem list was used for detecting the types of problems. The DT consists of 39 items grouped together in 5 domains: practical (6 items), family (4 items), emotional (6 items), spiritual/religious (1 items), and physical (22 items) domains. problem list items are all binary variables (yes/no). The instrument reliability (Cronbach’s *α*) was.92 in Chung et al’s (2014) study that measured BC survivors’ levels of distress,^[8]^ and.81 in the present study. We obtained permission to use the instrument by sending an email request to the NCCN and used the Korean Edition available from https://www.nccn.org.

1. Inventory of Functional Status-Cancer (IFS-CA)

Functional status was measured using the inventory of functional status-cancer (IFS-CA) developed by Tulman et al (1991)^[9]^ and adapted to the Korean context by Seo and Lee (1997).^[10]^ The IFS-CA is a 39-item questionnaire designed to measure the functional status of women with cancer, and consists of 4 subscales: household and family activities (15 items), social activities (6 items), self-care activities (10 items), and occupational activities (8 items). Each item is rated on a 4-point scale (1 = never, 4 = always). Each respondent’s average score is obtained after removing irrelevant items such as “driving” (for a woman who does not drive) or “care of pets” (for a woman who does not have a pet). Higher scores indicate a higher physical functional status except for items 22, 23, 25, 27, 28, 29, 31, 33, 34, and 36, in which lower scores indicate a higher functional status, reverse-scored. Cronbach’s *α* was .82 across the subscales and .74 in household and family activities, .82 in social activities, .56 in self-care activities, and .72 in occupational activities at the time of instrument development, and .74, .66, .52, and .82, respectively, in the study of Seo and Lee (1997).^[10]^

1. Menopause Rating Scale (MRS)

Menopausal symptoms were measured using the Korean version of the menopause rating scale (MRS) developed by Heinemann et al (2003).^[11]^ The MRS is an 11-item tool designed to assess the menopausal symptoms in 3 areas: somato-vegetative symptoms (4 items), psychological symptoms (4 items), and urogenital symptoms (3 items). Each item is rated on a 5-point scale (0 = not present, 4 = very severe), with the total score ranging from 0 to 44. The total score in the range of 0 to 4 is assessed as no/little, 5 to 7 as mild, 8 to 15 as moderate, and ≥ 16 as severe menopausal symptoms. The Cronbach’s *α* at the time of instrument development was.84. The tool is openly available, and the Korean version can be retrieved from http://www.menopause-rating-scale.info.

##### 3.4.3.2. Caregiver outcome.

1. Sociodemographic characteristics

Basic information about the caregiver (the main caregiver of the patient) included name, sex, age, relationship with the patient, contact information, same household with the patient or not, address, and direct care time.

(2) Primary outcome
  1) Health-related Quality of Life (HRQoL) assessment tools
    (1) EuroQol 5-Dimension 5-Level (EQ-5D-5L)
    (2) EuroQol-Visual Analogue Scales (EQ-VAS)
    (3) Secondary outcome
1) **Caregiver Burden Inventory (CBI**)

Caregiver burden was measured using the Korean version of the caregiver burden inventory (CBI) developed by Novak and Guest (1989),^[12]^ translated and revised by Jang^[13]^. The Korean version of the CBI (K-CBI) divides the caregiver burden into 6 domains: time-dependent burden (items 1–5), development and achievement burden (items 6–10), physical burden (items 11–14), social burden (items 15–19), emotional burden (items 20–24), and financial burden (items 25–29). Each of the 29 items is rated on a 5-point scale (1 = strongly disagree to 5 = strongly agree), with the total score ranging from 29 to 145. Higher scores indicate a greater caregiver burden.

1. Core Seven-Emotions Inventory-short form (CSEI-s)^[10]^
2. Oxford Happiness Questionnaire (OHQ)

Caregiver’s level of happiness was measured using the oxford happiness questionnaire (OHQ) developed by Hills and Argyle (2002),^[14]^ and translated by Choi and Lee (2004).^[15]^ This 29-item scale measures the degree of personal happiness in the domains of self-control, positive emotions, and confidence. Each item is rated on a 6-point scale (1 = strongly disagree to 6 = strongly agree). Negative questions were reverse-scored. Higher scores indicated a higher level of happiness. Cronbach’s *α* of the scale was.90 in the study of Choi and Lee (2004)^[15]^.

### 3.5. Patient and caregiver feedback on each session

#### 3.5.1. Mentalizing the rooms of mind (MRM).

Mentalizing the rooms of mind (MRM) is a projective test,^[16]^ 1 of the mindfulness and loving beingness psychotherapy techniques designed to help participants visualize and materialize their spontaneous (now and here) mental states ‐ unperceived thus far ‐ in a room structure.^[17]^ Through this technique, individuals can objectify the states of their mind in interactional relationships. It also helps consultants organize their vague states of mind through introspection. It is a useful tool with high clinical utility value in the evaluation and follow-up of mental states.

#### 3.5.2. Feedback assessment.

##### 3.5.2.1. Assessment plan.

1. Participant assessment

The outcomes for the assessment items were gathered twice from the participants: before the application of the program and after Session 8. Observational program assessment was performed during each session by means of MRM and interviews with the patient and caregiver. Table 2 outlines the study flow and assessment plan.

**Table 2:** Study Schedule Table.

	Period			Screening (Consent, enrollment)	Pre-assessment (Session 1)	Post-assessment (Session 8)
Results of inclusion/exclusion criteria				○		
Written consent form (reading and signing)				○		
Patient	Sociodemographic survey				○	
	Psychological testing				○	
	Primary outcome	HRQoL assessment scales	EQ-5D-5L		○	○
			EQ-VAS			
	Secondary outcome	Instruments for assessing pain, severity, and impairment of activities of daily living			○	○
		Numeric Pain Rating Scale (NPRS)			○	○
		Core Seven-Emotions Inventory-short form, CSEI-s)			○	○
		Pittsburgh Sleep Quality Index (PSQI)			○	○
Caregiver	Sociodemographic survey				○	
	Psychological testing				○	
	Primary outcome	HRQoL assessment scales	EQ-5D-5L		○	○
			EQ-VAS			
	Secondary outcome	Caregiver Burden Inventory (CBI)			○	○
		Core Seven-Emotions Inventory-short form, CSEI-s)			○	○
		Oxford Happiness Questionnaire (OHQ)			○	○

CBI = caregiver burden inventory, EQ-5D-5L = euroqol-5 dimension 5-level, EQ-VAS = euroqol visual analogue scale, HRQoL = health-related quality of life, OHQ = oxford happiness questionnaire.

1. Research team’s assessment (session report on the expert and participant feedback)

The researchers who guided the patient and caregiver through their respective programs listened to and recorded their feedback after each session. The principal researcher, the researchers who gathered feedback from the patient-caregiver pair, and experts involved in each session then gathered and provided feedback on the program administered during each session. They specifically converged opinions on the program’s shortcomings and items that needed improvement, as well as provided comprehensive feedback and estimated costs for future clinical application. Finally, the contents of the feedback were reflected in the program details for the next session, ensuring that continuous improvement occurred throughout the program period.

### 3.6. Data analysis

The assessment data were analyzed using quantitative and qualitative methods.

1. Quantitative analysis

The characteristics of the pain management and QoL questionnaires for the patient and caregiver were examined. Data for inferential statistical analysis were collected twice: once before Session 1 and again at the end of Session 8. The paired sample chi-square test was used to analyze the exploratory results of each questionnaire completed at baseline and after the program.

1. Qualitative analysis

The contents of the feedback interviews with the patient and caregiver conducted after each session, as well as the results of MRM testing of projected emotions, were analyzed qualitatively.

### 3.7. Ethical considerations

As this was an observational study, any risks other than those associated with the general treatment process are unlikely to occur. However, because mental and psychological risks cannot be ruled out during an interview, a therapist and a BC specialist in charge of the patient and caregiver were on call to intervene as needed.

The program used in this study was designed to be completed in 100 minutes, with a 10-minute break after 60 minutes to account for physical and mental fatigue. Furthermore, participants could request a break at any time during the program.

The information and data obtained from the research participants were protected and used under absolute anonymity guarantees, ensuring that their identities were never revealed under any circumstances. Furthermore, when the findings of his study were published, every precaution was taken to ensure that no personal information about the participants was disclosed anywhere in the published article.

All data collected and obtained during the study were used exclusively for research and report writing purposes. To prevent loss or theft, participants’ data were kept in the principal researcher’s archives under lock and key. All data will be destroyed and discarded after a 3-year retention period.

## 4. Discussion

BC is the most common cancer in Korean women, accounting for 20.5% of all female cancers in 2018, with the number of BC patients nearly doubling over a decade, rising from 12,823 in 2008 to 23,547 in 2018.^[18]^ Despite the rapid increase in its incidence, the survival rate of BC patients is much higher than that of other cancers, owing to advances in early detection and therapeutic advances in surgery, chemotherapy, adjuvant radiation therapy, and adjuvant hormonal therapy. The primary goal of cancer treatment used to be to increase cancer patients’ survival time. However, since the 2000s, the importance of their perceived well-being has been increasingly emphasized in cancer treatment, in light of the difficulties that BC patients face as a result of pain, depression, anxiety, reduced QoL, and psychosocial maladjustment such as sleep disturbances from the time of diagnosis.^[19]^ As a result, BC patients have a growing desire for treatment to extend survival while also minimizing side effects of cancer treatment and improving QoL. Cancer patients report high satisfaction with oriental medicine treatments in this regard,^[20]^ and previous studies have consistently reported the effectiveness of acupuncture treatment against hot flushes, pain, and fatigue in BC patients. Given the patients’ needs for high-quality healthcare services, it is past time to establish integrative healthcare services that leverage the benefits of western medicine, oriental medicine, and psychosocial services.

In this context, among the models developed for the application of integrative healthcare services to patients with the 4 major severe diseases and geriatric diseases, this study used the BC patients’ pain management and QoL enhancement model. In doing so, it aimed to provide basic data for the establishment of a healthcare service system for patients suffering from chronic pain due to diseases such as BC by systematically integrating previously unsystematically applied interventions, with only limited clinical data, into the current healthcare system and soliciting feedback from patients and caregivers.

This protocol was designed for a single case and is not suitable for gathering more diverse opinions. However, more effort will be expended in gathering detailed feedback from the patient and caregiver after each session. Furthermore, the program’s flexibility for a single case, which is advantageous over multiple-case programs, will be used to modify and improve the program as it progresses based on feedback received at each session for the next session. This has the advantage of determining which program was the most satisfactory and useful for improving QoL from the patient and caregiver’s perspectives. The findings of this study are expected to serve as the foundation for revising the integrative healthcare service model into a patient-centered model. The revised model will be used for future clinical pathway development research, allowing it to be used in clinical settings in any healthcare facility.

## Acknowledgments

This research was supported by a grant from the Korea Health Technology R&D Project through the Korea Health Industry Development Institute (KHIDI), funded by the Ministry of Health & Welfare, Republic of Korea.

## Author contributions

**Conceptualization:** MJC, WBH, HWK.

**Data curation:** MJC, WBH, HWK.

**Formal analysis:** MJC.

**Funding acquisition:** HWK.

**Investigation:** MJC.

**Methodology:** MJC.

**Project administration:** MJC.

**Supervision:** HWK.

**Writing-original draft:** MJC.

**Writing-review & editing:** MJC, HBC, UJC, HJW, YHH, HWK.
